# Ocean acidification reduces transfer of essential biomolecules in a natural plankton community

**DOI:** 10.1038/srep27749

**Published:** 2016-06-21

**Authors:** J. Rafael Bermúdez, Ulf Riebesell, Aud Larsen, Monika Winder

**Affiliations:** 1 GEOMAR | Helmholtz Centre for Ocean Research Kiel, Germany; 2 Facultad de Ingeniería Marítima, Ciencias Biológicas, Oceánicas y Recursos Naturales. Escuela Superior Politécnica del Litoral, ESPOL, Guayaquil, Ecuador; 3 The Hjort Centre for Marine Ecosystem Dynamics, Uni Research Environment, 5008 Bergen, Norway; 4 Department of Ecology, Environment and Plant Sciences, Stockholm University, Sweden

## Abstract

Ocean acidification (OA), a process of increasing seawater acidity caused by the uptake of anthropogenic carbon dioxide (CO_2_) by the ocean, is expected to change surface ocean pH to levels unprecedented for millions of years, affecting marine food web structures and trophic interactions. Using an *in situ* mesocosm approach we investigated effects of OA on community composition and trophic transfer of essential fatty acids (FA) in a natural plankton assemblage. Elevated *p*CO_2_ favored the smallest phytoplankton size class in terms of biomass, primarily picoeukaryotes, at the expense of chlorophyta and haptophyta in the nano-plankton size range. This shift in community composition and size structure was accompanied by a decline in the proportion of polyunsaturated FA (PUFA) to total FA content in the nano- and picophytoplankton size fractions. This decline was mirrored in a continuing reduction in the relative PUFA content of the dominant copepod, *Calanus finmarchicus*, which primarily fed on the nano-size class. Our results demonstrate that a shift in phytoplankton community composition and biochemical composition in response to rising CO_2_ can affect the transfer of essential compounds to higher trophic levels, which rely on their prey as a source for essential macromolecules.

Biomolecules synthesized by primary producers such as fatty acids (FA), and in particular polyunsaturated FA (PUFA), are considered essential metabolites for zooplankton and fish larvae^1^. As most consumers cannot synthesize these compounds *de novo*, they have to acquire them through their diet^1^. Laboratory experiments on the PUFA composition of microalgal cultures under different CO_2_ scenarios revealed a range of responses, ranging from increased^2^, to stable^3^, to reduced PUFA fractions^4^,^5^,^6^. Considering that food quality, including the FA composition, can be as important as food quantity for the transfer efficiency up the food web and the productivity of higher trophic levels^7^, a better understanding of OA effects on the biochemical composition of primary producers and the trophic transfer of this signal seems essential.

At community level, the food quality to a large extent is determined by the phytoplankton species composition, as different algal taxonomic groups have distinct biochemical signatures^8^. Experiments with natural plankton assemblages indicate that changing *p*CO_2_ can have pronounced impacts on phytoplankton community structure^9^,^10^, particularly small-sized algae like picoeukaryotes and cyanobacteria^11^,^12^. Thus, OA can affect the quality and quantity of food available for upper trophic levels by changing both the taxonomic composition and the biochemical signature of primary producers.

To investigate the effects of OA on phytoplankton species composition and consequences of biochemical transfer to upper trophic levels, we conducted an *in situ* mesocosm study enclosing a natural North Sea plankton community. The carbonate chemistry in the mesocosms was manipulated via the addition of CO_2_-saturated seawater to yield *p*CO_2_ ranging from current to projected levels by end of this century^13^, plus two extreme treatments beyond projected levels. We monitored the plankton community composition, FA content of three size fractions (micro: 100–10 μm, nano: 10–2.7 μm, pico: 2.7–0.3 μm), as well as zooplankton FA in the dominant copepod *Calanus finmarchicus* over the course of the experiment, and classified FA as PUFA, MUFA (monounsaturated fatty acids) and SFA (saturated fatty acids).

## Results and Discussion

**Figure 1:**
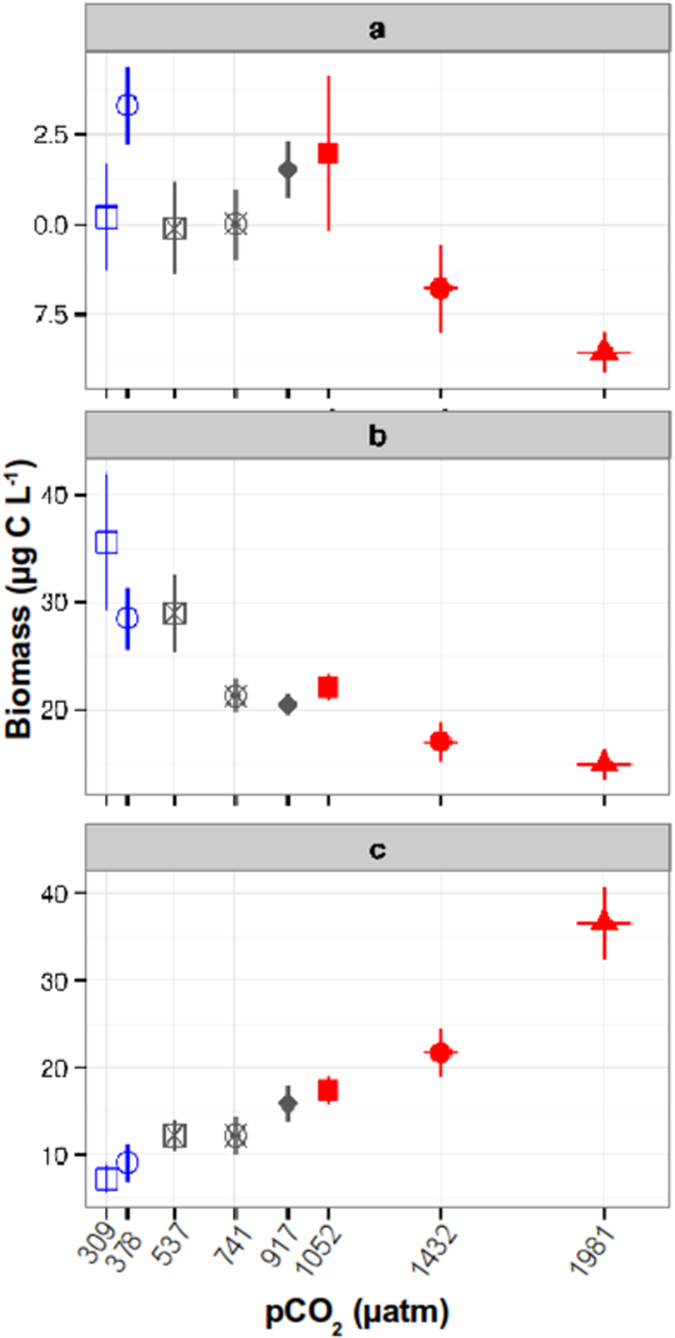
Average calculated biomass of the phytoplankton between sampling days 3 and 25, by size fraction: (**a**) microplankton (>10 μm), (**b**) nanoplankton (10–2.7 μm) and (**c**) picoplankton (<2.7 μm) in the CO_2_ gradient treatments during the experiment. A Mixed Effect Model analysis showed that all size fractions had significant differences in biomass between the *p*CO_2_ treatments. The x-axes display the mean CO_2_ levels (μatm) during the analyzed time period in each mesocosm, bars show standard error.

**Figure 2:**
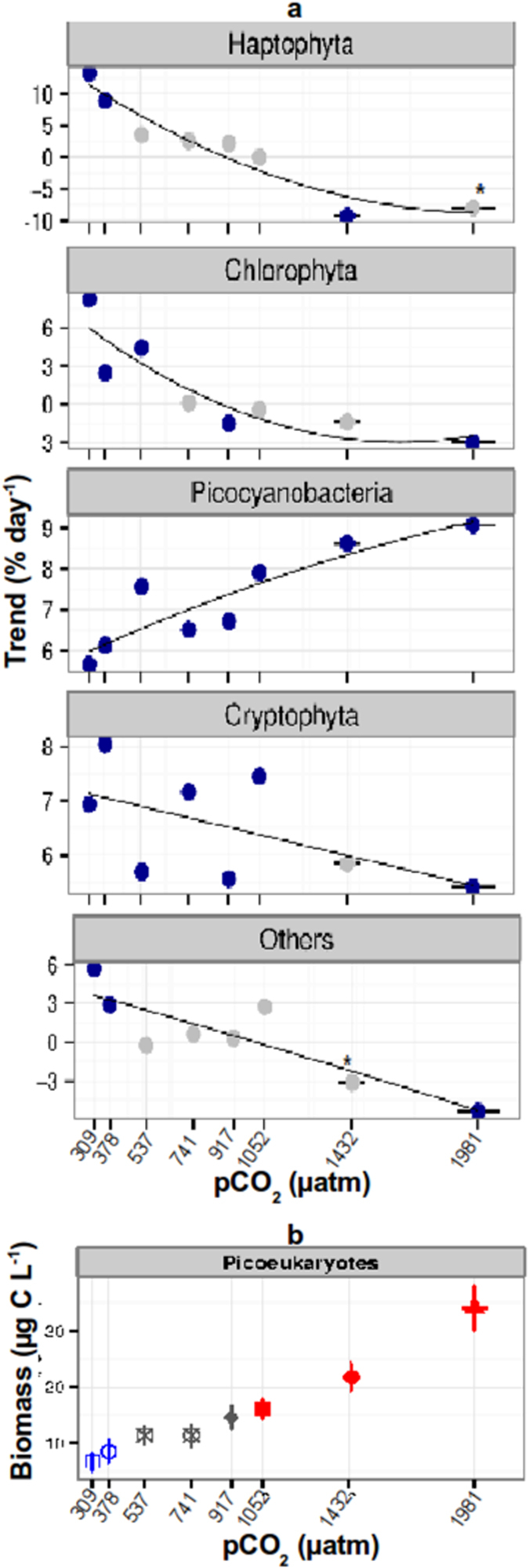
(**a**) Mann Kendall trend statistics of major phytoplankton taxonomic group biomass, between sampling days 3 and 25, across mesocosm treatments. Trends are shown as percent change per day (Trend %day-1); blue dots indicate significant trends (p < 0.05), grey dots no significant change (*p-value = 0.056). The x-axes display the mean *p*CO_2_ levels (μatm) during the analyzed time period in each mesocosm. (**b**) Mean of picoeukaryote biomass between day 3 and 25 during the experiment. A Mixed Effect Model analysis revealed a significant increase at elevated *p*CO_2_ levels in the biomass between the different treatments (*p*CO_2_: F = 83.2834, p < 0.0001, df = 84; time: p = 0.360; *p*CO_2_: time interaction, p = 0.102). Man Kendall trend test could not be applied to this group, as biomass increased non-monotonic during the experiment. Bars show standard error.

The size structure of the phytoplankton community shifted during the experiment in the different CO_2_ treatments. A Mixed Effect Model (MEM) analysis from sampling day 3 to sampling day 25 performed by size fraction revealed that plankton biomass in the micro- (Fig. 1a, Fig. S1) and nano-size (Fig. 1b, Fig. S1) fractions changed significantly through time at different *p*CO_2_ treatments (MEM micro: *p*CO_2_ F = 12.95, p < 0.0001, df = 85; time F = 24.32, p < 0.0001, df = 85; *p*CO_2_: time interaction F = 12.09, p < 0.0001, df = 85. MEM Nano: *p*CO2 F = 28.73, p < 0.000, df = 81; time F = 5.17, p = 0.025, df = 81; *p*CO_2_: time interaction F = 42.37, p < 0.0001, df = 81), while the pico-size fraction biomass (picocyanobacteria and picoeukaryotes) showed a fast change with *p*CO_2_, reaching a significantly higher abundance at elevated treatments in a short time (pico: *p*CO_2_ F = 74.47, p < 0.0001, df = 85; time p = 0.8496; *p*CO_2_: time interaction F = 3.6873, p = 0.0582) (Fig. 1c; Fig. S1). These observations are consistent with some studies showing that small-sized phytoplankton benefit from high *p*CO_2_ levels in terms of growth rate and biomass accumulation^11^,^12^. A Mann-Kendall trend analyses over the duration of the experiment revealed that this change was due to differential responses of major taxonomic groups to elevated *p*CO_2_ (Fig. 2a). Chlorophyta (mostly Prasinophyecea in the nano-size range), the most abundant group, gradually increased significantly (*p* < 0.05) in low *p*CO_2_ treatments and did not change at intermediate and high *p*CO_2_ conditions over the experimental duration (Fig. 2a). Haptophyta, dominated by the coccolithophore *Emiliania huxleyi* in the nano-size class, increased significantly at low *p*CO_2_ levels, were unaffected at intermediate values and significantly declined at high CO_2_ treatments (Fig. 2a). The negative CO_2_ effect on the coccolithophores observed in the present experiment is consistent with other mesocosm studies that showed a diminished abundance of calcifying algae at high *p*CO_2_ levels attributed to reduced growth rates at increased CO_2_ conditions^14^. The same pattern was observed for the sum of miscellaneous phytoplankton taxa (Euglenophyta, Heterokontophyta and Dinophyta in the micro-size range) (Fig. 2a, Others), which comprised only a small fraction of algal biomass in all mesocosms. Cryptophyta in the nano-size fraction increased significantly over time with no consistent pattern between *p*CO_2_ treatments (Fig. 2a). Picocyanobacteria (*Synechococcus*) increased significantly over the course of the experiment in all mesocosms with a stronger increase at high *p*CO_2_ but represented a small fraction of the total biomass (Fig. 2a, Fig. S1). Picoeukaryote biomass, as indicated by a mixed model effect, increased with the treatment gradient and became the most dominant taxa at elevated *p*CO_2_ towards the end of the experiment (Fig. 2b, Fig. S1). The change of the community composition in terms of species biomass over time and CO_2_ was confirmed by a Non-metrical Multidimensional Scaling analysis, which showed a strong gradual shift (Fig. S2) confirmed by an Analysis of Similarity (R = 0.40; p < 0.001), while the change associated with CO_2_ was comparatively smaller although significant (R = 0.18; p < 0.001).

**Figure 3:**
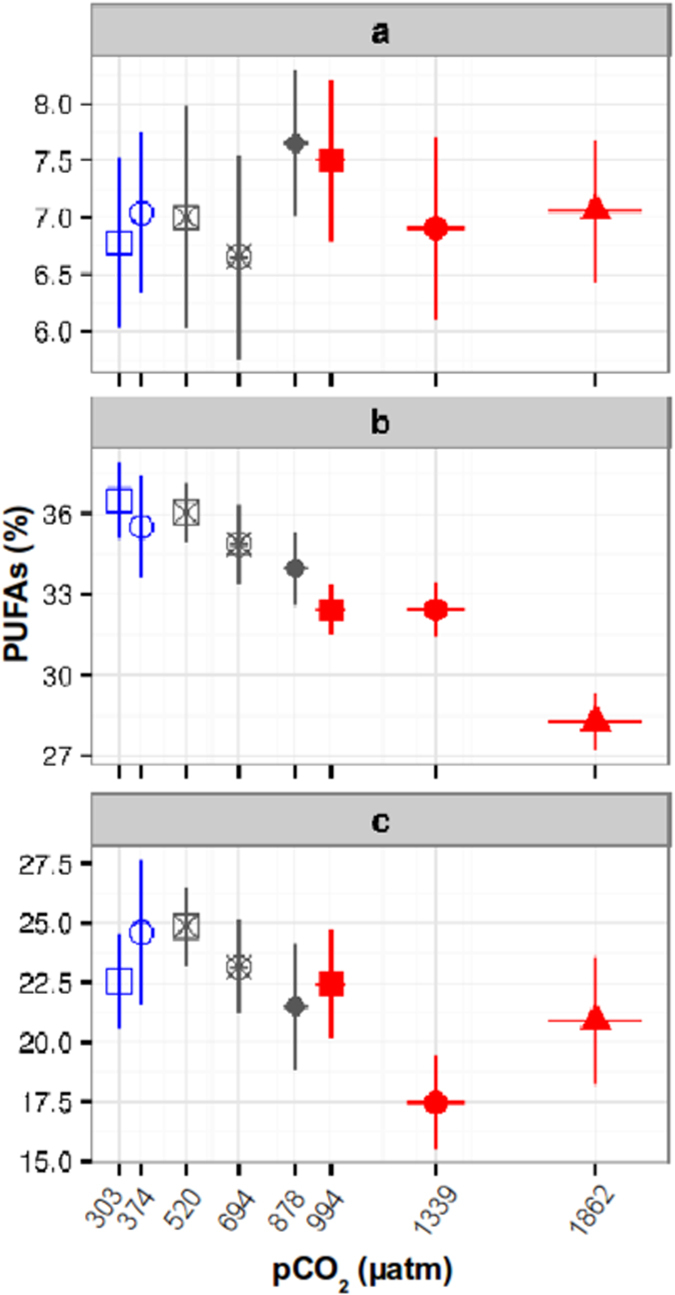
Relative Polyunsaturated Fatty Acids (PUFAs) content in (**a**) microplankton (>10 μm; no significant difference), (**b**) nanoplankton (10–2.7 μm; MEM, F = 14.70, p < 0.001, df = 44) and (**c**) picoplankton (<2.7 μm; MEM, F = 6.58, p = 0.013, df = 42) size fractions over the *p*CO_2_ gradient treatments between sampling days 5 and 29. The x-axes display the mean *p*CO_2_ levels (μatm) during the analyzed time period in each mesocosm. Bars show standard error.

The change in the plankton community composition was accompanied with a change in the FA composition of the different plankton size fractions. The relative content of the ecologically important PUFA group revealed marked differences between size fractions, with ~7% (3 ± 2 ng L^−^¹) of total FA in the micro-, ~34% (39 ± 14 ng L^−^¹) in the nano- and ~20% (21 ± 15 ng L^−^¹) in the pico-size fraction. The PUFA content of the micro-size plankton fraction did not change with *p*CO_2_ (Fig. 3a), while there was a significant negative *p*CO_2_ effect at the nano- and pico-size fraction (Fig. 3b,c) at low *p*CO_2_ levels. In the pico and nano- size fractions the most important PUFA where Eicosapentaenoic (20:5n3, EPA), Eicosatrienoic (20:3n3, ETE) and Docosahexaenoic (22:6n3, DHA) acids (Fig. S3a,b), representing ~60% of the total PUFA. The CO_2_-related PUFA decrease in the nano- and pico-size fractions was associated with an increase of both SFA and mostly MUFA (Fig. S4).

The reduced PUFA content in the nano-size fraction is likely a result of the change in plankton community composition, especially due to the reduced Haptophyta biomass at high *p*CO_2_ level, which are a very good sources of PUFA^8^. The reduced PUFA content in the pico-size fraction can be due to an increase of picocyanobacteria and picoeukaryotes abundance. The first are known for being a poor PUFA source^15^, while the high abundance of the later at elevated *p*CO_2_ suggests a PUFA loss in this group. Although we have not identified the species composition of the picoeukaryote group, significant increases of pico- chlorophyta (Prasinophyecea) have been observed in plankton assemblages at elevated CO_2_ conditions^11^. Chlorophyta have a relatively low PUFA content^8^, which may contribute to the reduced PUFA content at high *p*CO_2_. Given that our experiment did not allow the distinction between an indirect effect on PUFA concentration through shifts in species composition and a direct CO_2_ response on cell physiology, the latter cannot be excluded. It has been shown for both, Chlorophyta and the Haptophyte *Emiliania huxleyi* that culturing at high CO_2_ reduced their PUFA content^4^,^16^. This suggests that the relative PUFA decrease in both size fractions is most likely a combination of a change in community composition and a direct CO_2_ effect on cell physiology.

Our results showing low PUFA levels at high *p*CO_2_ are contrasting the response of an Arctic plankton community, in which relative PUFA content increased at elevated *p*CO_2_ levels^17^. The divergent results can be attributed to the different plankton community compositions^8^. However, this can also be due to the size-fractionation of the samples for FA analysis in the present experiment, which was not done in in the Arctic study. A change in the FA of a single species within the community, especially if it has a large biomass, may mask changes in other size classes or taxonomic groups. The observed PUFA increment in the Arctic was associated to an elevated abundance of large-sized dinoflagellates^17^, which are typically high in PUFA content^8^ and therefore may not reflect a net positive CO_2_ effect on all primary producers, but rather in this specific group. Thus, biochemical changes of primary producers belonging to different size fractions may affect higher trophic levels differently as aquatic predators are size-selective^18^.

**Figure 4:**
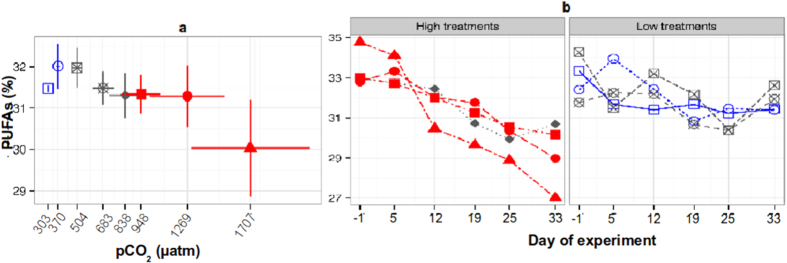
(**a**) Relative Polyunsaturated Fatty Acids (PUFAs) content in the copepod *Calanus finmarchicus* across the different CO_2_ treatments (MEM, F = 80.74, p = 0.001, df = 31). The x-axes display the mean *p*CO_2_ levels (μatm) between days 5–33 in each mesocosm. Bars show standard error. (**b**) Relative PUFAs content of *C. finmarchicus* between days 1–33 across CO_2_ treatments. High *p*CO_2_ treatments (left) showed a significant decrease through time (Linear regression, 1120: R^2^ = 0.73, p = 0.017; 1400: R^2^ = 0.97, p = <0.001; 2000: R^2^ = 0.86, p < 0.01; 3000: R^2^ = 0.93, p = 0.001).

The decrease of PUFA in the nano-size fraction observed in this study affected the PUFA profile of the dominant copepod species. The PUFA content in *C. finmarchicus* represented ~32% (120 ± 40 ng ind.^−^¹) of total FA, and the relative PUFA content decreased significantly with higher *p*CO_2_ levels (Fig. 4a). While staying constant at low *p*CO_2_ levels, it gradually and significantly decreased at *p*CO_2_ treatments of 1120 μatm and higher, with an average loss of ~4%, and strongly declined (~10%) at the highest *p*CO_2_ treatment over the course of the experiment (Fig. 4b). In the copepod, like in the plankton size fractions, the Eicosapentaenoic (20:5n3, EPA), Eicosatrienoic (20:3n3, ETE) and Docosahexaenoic (22:6n3, DHA) acids where the most abundant (Fig. S3c) and represented around ~90% of the total PUFA. The relative PUFA decline was associated with an increase of MUFA and SFA (Fig. S5). The ratio of the different FA classes (PUFA, MUFA, and SFA) in copepods closely resembled the corresponding proportions in the nano-size phytoplankton fraction (Table 1). This close resemblance was maintained throughout the entire experiment (Table S1), indicating that *C. finmarchicus* strongly relied on this size fraction as a PUFA resource. The copepod’s prey-dependent FA profile is coherent with previous studies showing that copepods strongly rely on their diet for essential PUFA and that their FA composition mirrors the algae they graze on^19^, and with reports showing that this species capitalizes efficiently on small-sized algal prey when present in high amounts, like in the present study^20^,^21^. Even though we were not able to evaluate the impact of the PUFA reduction on *C. finmarchicus* life cycle (~1 year long)^21^ as the time frame of the experiment (~6 weeks) did not allow the observation of significant changes in the population structure; it has been observed in previous studies that even small changes in the food source of FA have important consequences in copepods and fish^19^,^22^.

Although a direct CO_2_ effects on copepod FA synthesis cannot be excluded, it seems unlikely. Previous experiments showed that *C. finmarchicus* is rather insensitive to elevated CO_2_ exposure^23^. Another factor that may have influenced FA transfer to the copepods in the present study is the shift in size of the plankton community, with the pico-size fraction becoming more dominant at high *p*CO_2_ levels. A reduction of mean prey size towards small-sized cells can cause a decline in feeding efficiency of large copepods, such as *C. finmarchicus,* irrespective of their feeding mechanism^18^. Therefore, the observed decrease in the *C. finmarchicus* PUFA content at high *p*CO_2_ conditions may be a combined consequence of a CO_2_-driven deterioration of the food quality and prey availability.

Our study provides the first evidence that a CO_2_-driven shift in community composition and associated change in food quality in terms of FA at the base of the food web can be transferred to primary consumers in a natural marine assemblage. A diatom grown at 750 μatm *p*CO_2_ showed a PUFA decrease of ~20% that, when used to feed a copepod, produced a ~29% PUFA reduction in them with a concomitant decline in both somatic growth and egg production of ~85%^19^. For fish it has been shown that a decrease of just 0.6% in the amount of n-3 FA in the food source can reduce their egg viability by ~10% and larval survival by ~15%^22^. Given that fish is a critical natural resource^24^, acidification-driven FA quality deterioration may reduce the availability of essential PUFA in human diets^25^. Nonetheless, the plankton community response to OA will strongly depend on how changes in CO_2_ affect species composition of primary producers, whereby genetic and physiological diversity in natural assemblages may buffer adverse CO_2_ effects^17^,^26^.

## Methods

An *in situ* mesocosm CO_2_ perturbation experiment in Raunefjord, southern Norway was performed during late spring 2011 using nine enclosures with a length of 25 m containing ~75 m^3^ of natural seawater. The mesocosms were set up and manipulated as described in detail by Riebesell *et al.*^27^. The carbonate system was manipulated through the addition of CO_2_-saturated seawater to seven of the nine mesocosms in four steps between days 0 to 3 to achieve target values of 380, 560, 840, 1120, 1400, 2000 and 3000 μatm plus one control unit with a natural concentration of 280 μatm. Samples for the calculation of the *p*CO_2_ in each mesocosm were taken every sampling day, and was calculated based on measurements of total alkalinity, pH (in total scale), salinity and temperature, using the computer program CO2SYS^28^. Samples for total alkalinity measurements were filtered on a Whatman GF/F filter and measured by open-cell acidimetric titration as describes by Dickson (2010)^29^. pH was measured spectrophotometrically with a VARIAN Cary 100 in 10 cm cuvette at 25°C and then recalculated to *in-situ* temperature as described in Dickson (2010)^29^. The precision on the pH measurements was typically over 0.001 units at high and 0.002 units at low pH. Vertical profiles of temperature and conductivity were taken daily in each mesocosm with a CTD60M (Sun and Sea Technologies). The measurements of *p*CO_2_, pH and inorganic nutrients for all nine mesocosm are provided in Table S2.

On day 14, nitrate and phosphate were added to all mesocosms to a final concentration of 5 and 0.2 μmol l^−1^, respectively. Samples for phytoplankton counts and flow cytometry were taken every second day and for fatty acids every fourth day using a depth-integrated water sampler (Hydrobios, Kiel, Germany) covering the upper 20 m of the water column from days 1 to 25; integrated zooplankton net tows were taken every 7th day starting the day before the CO_2_ manipulation (day-1) until day 33. Phytoplankton cell counts were carried out from 50 ml sample water, fixed with alkaline Lugol’s iodine (1% final concentration) using an Utermöhl chamber with an inverted microscope (ZEISS Axiovert 100) and identified up to species level when possible (Table S3). Picoplankton cells were enumerated using a bench-top Becton Dickinson FACSCalibur flow cytometer (FCM) equipped with a 488 nm argon laser. Biovolume was calculated according to geometric shapes and converted to cellular organic carbon using taxon-specific conversion equations; for micro- and nanophytoplankton according to Menden-Deuer^30^, and for picophytoplankton according to Worden *et al.*^31^. For algal FA, 1 L of seawater was filtered in three size fractions: 100 to 10 μm (micro), 10 to 2.7 μm (nano) and 2.7 to 0.3 μm (pico) by using, non-combusted Millipore NY1002500 (10 μm ø pore), Whatman GF/D (2.7 μm ø pore) pre-combusted (450 °C, 6 h) and Advantec Grade GF 75 (0.3 μm ø pore) filters, respectively. Individuals of the copepod *Calanus finmarchicus* (copepodite stage V) were sorted for FA measurements. All samples were immediately stored at −80°C until analysis. FA were measured by gas chromatography as fatty acid methyl esters (FAME) following Klein Breteler *et al.*^32^. FAME were analyzed by a Thermo GC Ultra gas chromatograph equipped with a non-polar column (RXI1-SIL-MS 0.32 μm, 30 m, company Restek) and Flame ionization detector and were clustered according to their degree of saturation: saturated (SFA), monounsaturated (MUFA) and polyunsaturated (PUFA).

To identify differences in the relative content of FAs between the treatments a nested Mixed Effects Model (MEM) was used to determine the differences in relative fatty acid content (%) between the CO_2_ treatments (μatm) through time, with the treatment level as nested random variable (random distribution of CO_2_ treatments among the mesocosm). Mann-Kendall trend test was applied to analyze for monotonic trends in the data to determine the temporal change for plankton taxonomic groups in terms of calculated biomass throughout the experiment. Slopes were expressed as % day^−1^ by dividing the experimental mean of the variable; in the case of the picoeukaryotes, where the biomass distribution was non-monotonic, MEM analysis as described above was performed. The similarity in the structure of the plankton community between the treatments in terms of calculated species biomass in the mesocosm was analyzed by Non Metrical Multidimensional Scaling (NMDS) with Bray distance, auto-transformation and 4 dimensions (k = 4). An analysis of similarity (ANOSIM) using a Bray-Curtis distance matrix and 5000 permutations was used to corroborate the NMDS results. All statistical analyses were done using the R software environment 3.0.1^33^.

## Additional Information

**How to cite this article**: Bermúdez, J. R. *et al.* Ocean acidification reduces transfer of essential biomolecules in a natural plankton community. *Sci. Rep.* **6**, 27749; doi: 10.1038/srep27749 (2016).

## Supplementary Material

Supplementary Information

## Figures and Tables

**Table 1:** Ratios of FA in the copepod *Calanus finmarchicus* to different phytoplankton size fractions (micro: 10–100 μm, nano: 2.7–10 μm, pico: 0.3–2.7 μm).

	SFA:PUFA	sd	SFA:MUFA	sd	MUFA:PUFA	sd
*Calanus*	**1.67**	**0.14**	**3.48**	**1.03**	**0.48**	**0.1**
Micro-fraction	12.13	4.32	5.74	2.1	2.3	0.96
Nano-fraction	**1.41**	**0.33**	**3.19**	**1.37**	**0.52**	**0.22**
Pico-fraction	3.2	1.24	3.1	1.36	1.12	0.54

Ratios are shown for different FA classes: Saturated (SFA), Monounsaturated (MUFA) and Polyunsaturated (PUFA). Bold values show the similarity between *C. finmarchicus* and the nano-size phytoplankton fraction.
