# Supplementary material for: Ocean acidification reduces transfer of essential biomolecules in a natural plankton community
Source: Sci Rep. 2016 Jun 21;6:27749. doi: 10.1038/srep27749 (PMC4914976; doi:10.1038/srep27749)
Supplement: Supplementary Information [file srep27749-s1.pdf]

Supplementary Information

Ocean acidification reduces transfer of essential biomolecules in a natural plankton community

J. Rafael Bermúdez[1,2]*, Ulf Riebesell[1] , Aud Larsen[3], Monika Winder[1,4]

[1]GEOMAR | Helmholtz Centre for Ocean Research Kiel, Germany.

[2]Facultad de Ingeniería Marítima, Ciencias Biológicas, Oceánicas y Recursos Naturales. Escuela Superior Politécnica del Litoral, ESPOL, Guayaquil, Ecuador.

[3]The Hjort Centre for Marine Ecosystem Dynamics, Uni Research Environment, 5008 Bergen, Norway.

[4]Department of Ecology, Environment and Plant Sciences, Stockholm University, Sweden.

*Contact: jrbermud@espol.edu.ec

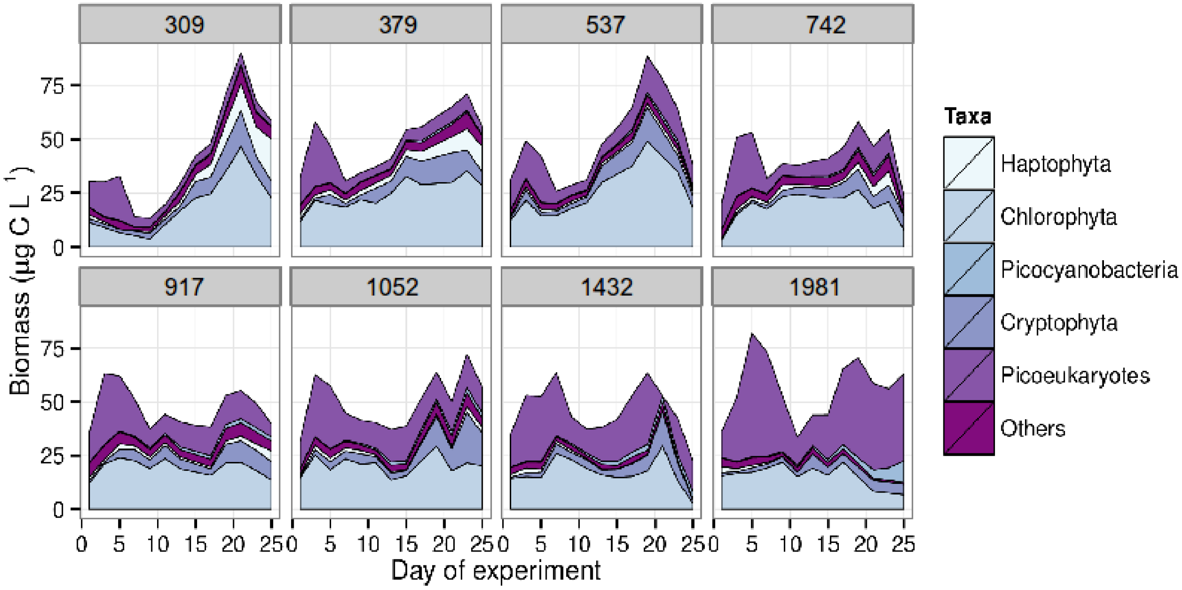

Figure S1: Plankton biomass of the dominant taxonomic groups in the different CO_2 treatments. Each treatment is labelled with its target CO_2 level (top, µatm). Nutrients were added at day 14. Phytoplankton biomass displayed a short-lived small bloom at the start of the experiment and a more pronounced bloom after nutrient addition.

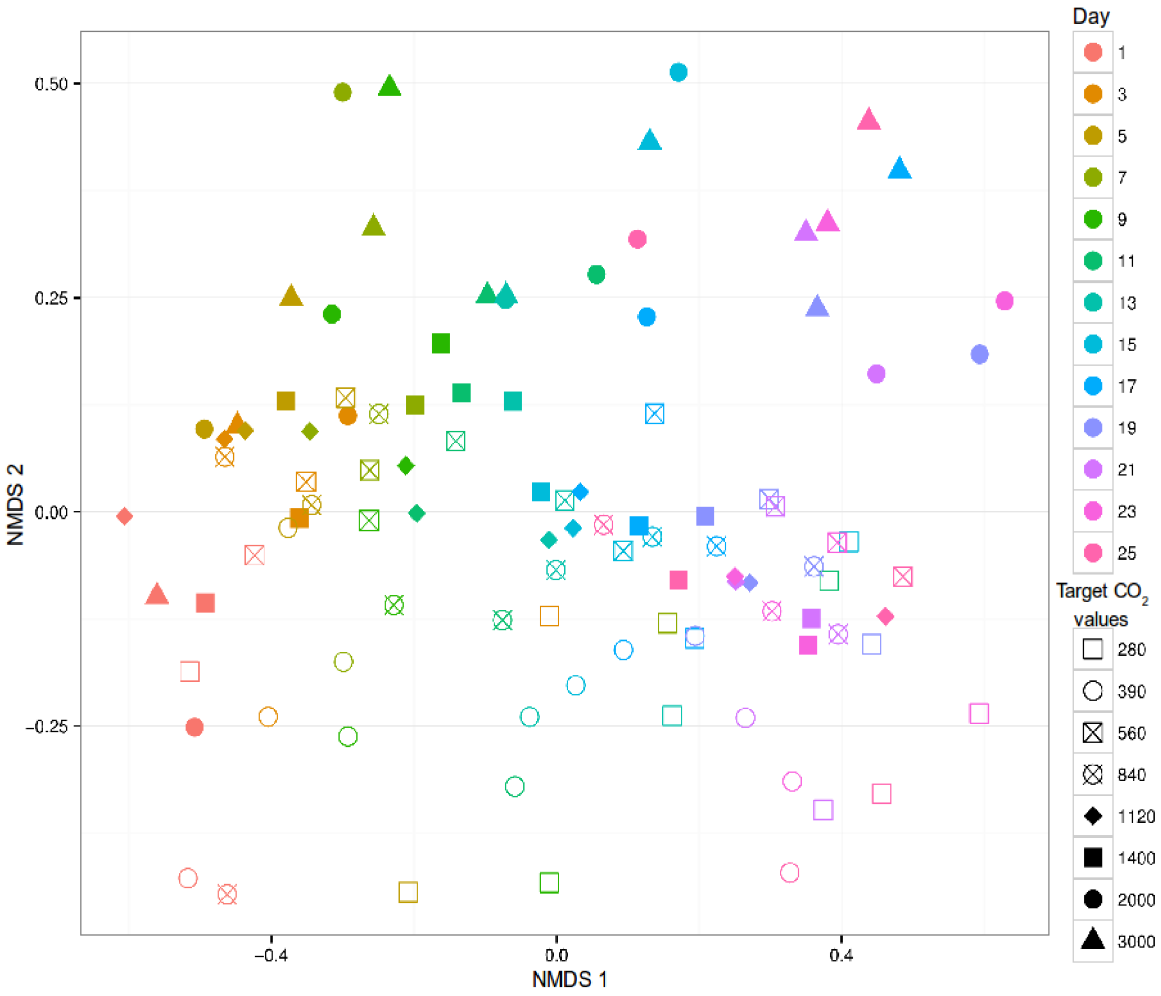

Figure S2: Non Metrical Multidimensional Scaling (NMDS) of the plankton community composition in terms of calculated biomass change through sampling days and CO_2 treatment. The NMDS 1 axis show that the phytoplankton communities strongly diverge through time, while the NMDS 2 axis show that the communities split between high (top) and low (bottom) CO_2 treatment levels. An Analysis of Similarity showed a significant difference on community composition between days (ANOSIM statistic R: 0.4, p= 0.0002) while the analysis between CO_2 treatments although significant was weaker (ANOSIM R: 0.17, p= 0.0002).

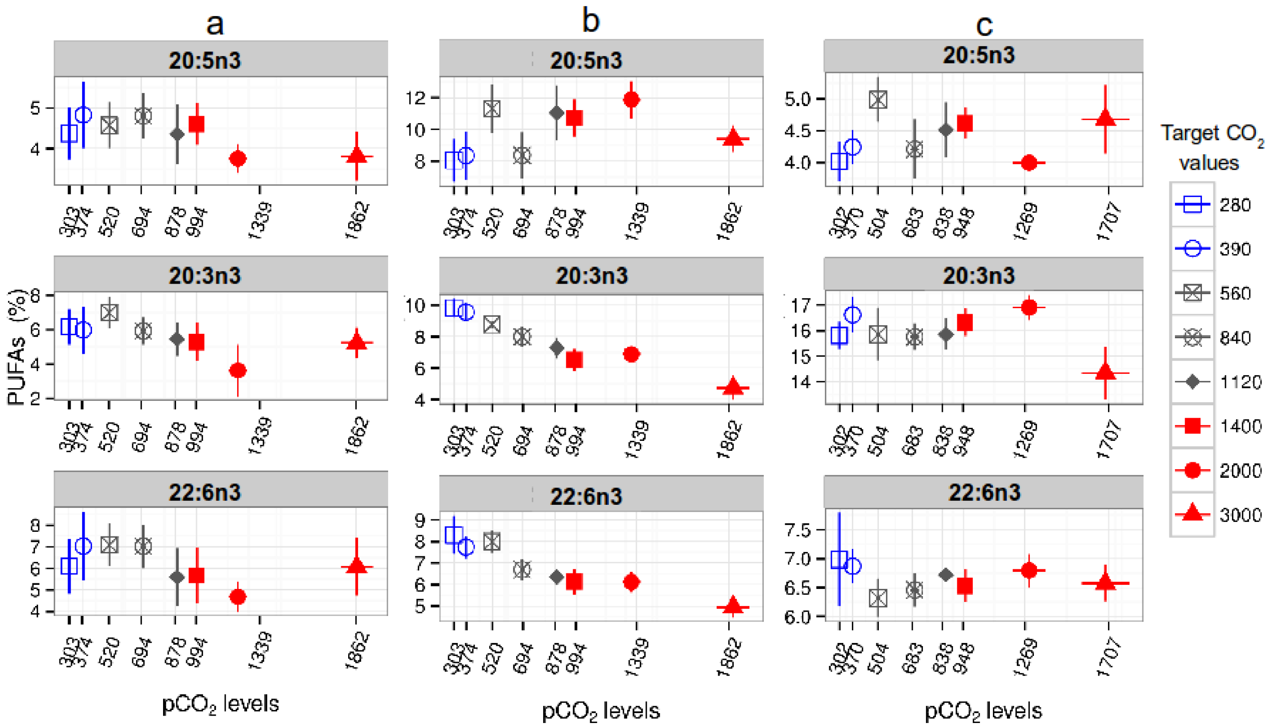

Figure S3: Relative content of the most abundat PUFA in the pico-size (a), nano-size (b) and *Calanus finmarchicus* (c) during the experiment (pico-size: 0.3-2.7 µm, nano-size: 2.7-10 µm) in the different *p*CO_2 treatments during the experiment. A mixed effect model analysis (MEM) showed that 20:5n3 presented no CO_2 related effect in any group ($p>0.05$). The 20:3n3 showed a CO_2 effect in the nano size fractions (MEM, $F=10.33$, $p<0.05$) and *C. finmarchicus* (MEM, $F=8.21$, $p<0.05$). The 22:6n3 showed a significant CO_2 effect only in the Nano-size fraction (MEM, $F=11.86$, $p< 0.05$).

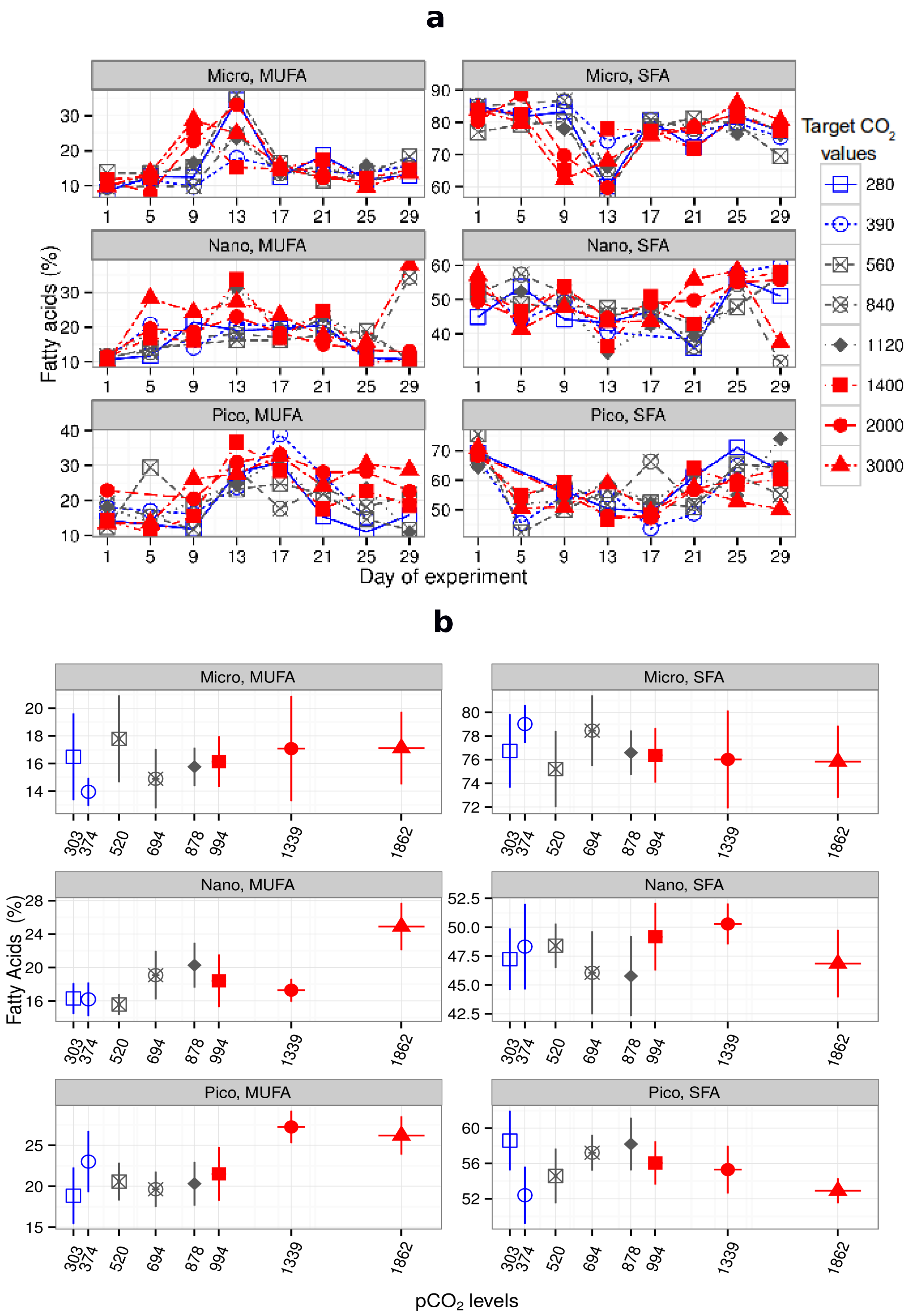

Figure S4: Relative plankton MUFA and SFA content during the experiment in different size fractions (micro: 10-100 µm, nano: 2.7-10 µm,pico: 0.3-2.7 µm) a)

through time, and b) in the different pCO_2 treatments.

a

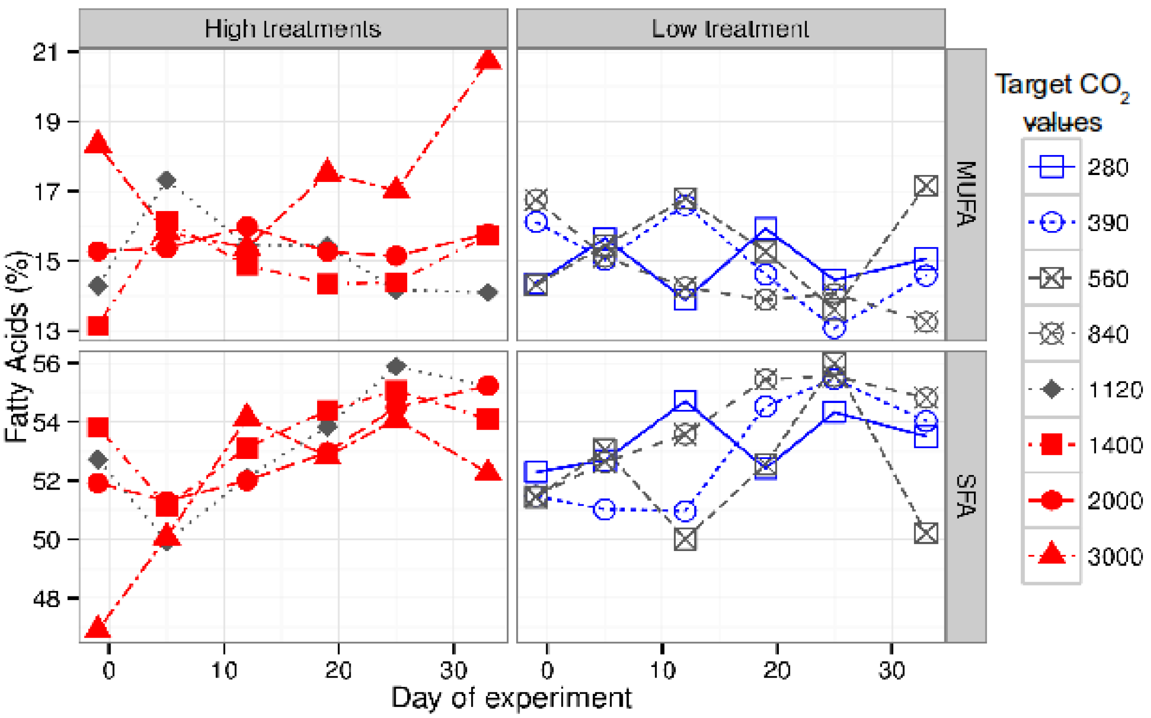

b

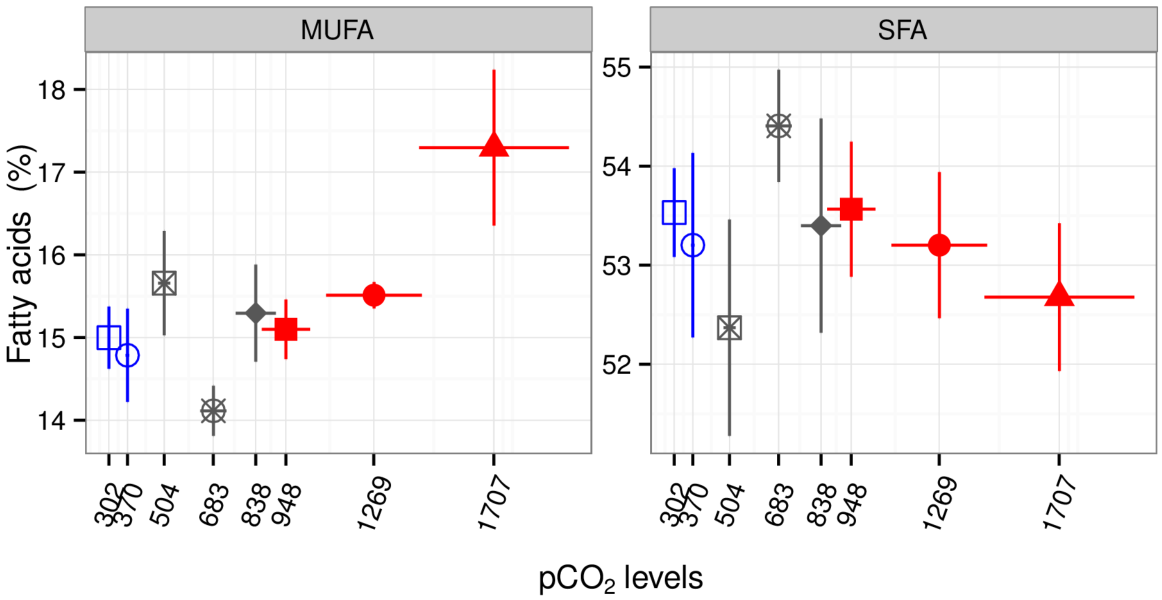

Figure S5: Relative MUFA and SFA content in the copepod *Calanus finmarchicus* a) through time, and b) in the different pCO_2 treatments.

Table S1: Ratios of different FA classes of the copepod *Calanus finmarchicus* and to different phytoplankton size fractions (micro: >10 µm, nano: 2.7-10 µm,pico: <2.7 µm) at day 1 and day 25. Ratios are shown for Saturated (SFA), Monounsaturated (MUFA) and Polyunsaturated (PUFA) fatty acids. Values in bold font type highlighted the similarity between *C. finmarchicus* and the nano-size phytoplankton fraction.

**Day 1**	SFA:PUFA	sd	SFA:MUFA	sd	MUFA:PUFA	sd
Calanus	**1.56**	**0.12**	**3.58**	**0.28**	**0.44**	**0.04**
Micro-fraction	14.69	3.7	8.05	1.39	1.75	0.45
Nano-fraction	**1.38**	**0.21**	**4.67**	**0.54**	**0.29**	**0.02**
Pico-fraction	5.06	1.37	4.35	1.06	1.25	0.59
**Day 25**						
Calanus	**1.81**	**0.04**	**3.82**	**0.33**	**0.48**	0.05
Micro-fraction	13.28	3.01	6.79	1.25	1.95	0.16
Nano-fraction	**1.74**	**0.26**	**4.45**	**1.09**	**0.41**	**0.12**
Pico-fraction	3.44	0.78	3.43	1.58	1.19	0.62

Table S2: Physical variables measured during the experiment when FA seston samples were taken. Nitrate, phosphate, silicate and ammonia are given in µmol l^{-1} and pH in total scale.

Mesocosm	Target CO_2	Day	Temperature (°C)	pCO2 (µatm)	pH	Nitrate	Phosphate	Silicate	Ammonia
M1	840	1	8.57	464.39	7.73	1.70	0.16	1.13	0.20
M1	840	5	9.47	891.40	7.50	0.49	0.08	0.80	0.05
M1	840	9	9.24	810.33	7.53	0.20	0.06	0.50	0.08
M1	840	13	9.84	745.51	7.56	0.20	0.07	0.45	0.13
M1	840	17	9.72	711.52	7.59	4.31	0.15	0.34	0.10
M1	840	21	9.68	623.95	7.64	1.90	0.04	0.04	0.07
M1	840	25	9.75	606.31	7.65	1.50	0.05	0.08	0.20
M1	840	29	NA	535.99	7.65	1.30	0.06	0.06	0.34
M3	1120	1	8.62	453.71	7.74	1.30	0.14	1.07	0.19
M3	1120	5	9.52	1168.60	7.41	0.39	0.06	0.77	0.08
M3	1120	9	9.25	1042.56	7.47	0.10	0.05	0.50	0.05
M3	1120	13	9.84	920.16	7.50	0.10	0.05	0.41	0.05
M3	1120	17	9.71	848.38	7.53	4.41	0.13	0.26	0.10
M3	1120	21	9.69	762.68	7.56	2.40	0.04	0.03	0.07
M3	1120	25	9.74	742.88	7.57	1.90	0.08	0.08	0.12
M3	1120	29	NA	621.29	7.58	1.80	0.07	0.06	0.36
M4	280	1	8.62	323.94	7.86	1.60	0.15	1.13	0.18
M4	280	5	9.52	300.03	7.89	0.59	0.08	0.80	0.12
M4	280	9	9.25	304.92	7.89	0.31	0.07	0.56	0.13
M4	280	13	9.85	319.20	7.88	0.10	0.06	0.52	0.16
M4	280	17	9.72	319.71	7.88	4.71	0.19	0.51	0.12
M4	280	21	9.69	299.58	7.90	2.50	0.05	0.18	0.10
M4	280	25	9.73	292.62	7.91	2.20	0.09	0.22	0.25
M4	280	29	NA	284.07	7.92	1.50	0.09	0.16	0.38
M5	1400	1	8.62	471.56	7.72	1.50	0.16	1.16	0.26
M5	1400	5	9.51	1324.86	7.33	0.49	0.08	0.80	0.05
M5	1400	9	9.28	1227.31	7.38	0.10	0.06	0.48	0.07
M5	1400	13	9.84	1101.05	7.43	0.10	0.04	0.35	0.10
M5	1400	17	9.72	967.45	7.48	4.31	0.12	0.16	0.12
M5	1400	21	9.69	854.84	7.52	3.20	0.06	0.02	0.19
M5	1400	25	9.74	826.70	7.54	2.00	0.07	0.07	0.20
M5	1400	29	NA	662.00	7.54	1.70	0.07	0.05	0.35
M6	390	1	8.62	373.88	7.81	1.50	0.17	1.11	0.22
M6	390	5	9.52	394.11	7.80	0.49	0.08	0.79	0.06
M6	390	9	9.26	392.27	7.81	0.20	0.07	0.62	0.11
M6	390	13	9.83	393.84	7.81	0.10	0.05	0.57	0.13
M6	390	17	9.72	390.08	7.81	4.31	0.13	0.43	0.14
M6	390	21	9.71	355.85	7.85	2.60	0.06	0.19	0.07
M6	390	25	9.73	353.47	7.85	1.80	0.04	0.20	0.23
M6	390	29	NA	334.87	7.84	1.40	0.04	0.13	0.24
M7	2000	1	8.62	454.67	7.74	1.30	0.15	1.07	0.21
M7	2000	5	9.51	2065.92	7.20	0.29	0.07	0.79	0.03
M7	2000	9	9.25	1746.04	7.25	0.10	0.05	0.51	0.05
M7	2000	13	9.83	1539.45	7.31	0.10	0.04	0.42	0.07
M7	2000	17	9.72	1260.96	7.38	4.31	0.13	0.24	0.03
M7	2000	21	9.71	1133.00	7.42	2.10	0.04	0.03	0.12
M7	2000	25	9.73	922.60	7.49	1.90	0.04	0.09	0.22
M7	2000	29	NA	753.07	7.50	1.50	0.04	0.02	0.23
M8	560	1	8.62	465.18	7.73	1.20	0.18	1.04	0.06
M8	560	5	9.53	594.55	7.65	0.39	0.07	0.79	0.03
M8	560	9	9.26	568.96	7.67	0.20	0.09	0.63	0.11
M8	560	13	9.85	570.57	7.67	0.10	0.07	0.55	0.11
M8	560	17	9.72	532.43	7.70	4.41	0.15	0.42	0.08
M8	560	21	9.72	484.37	7.73	2.60	0.05	0.12	0.03
M8	560	25	9.73	466.94	7.74	1.70	0.05	0.15	0.19
M8	560	29	NA	442.62	7.74	1.30	0.05	0.11	0.24
M9	3000	1	8.63	468.91	7.72	1.30	0.16	1.08	0.05
M9	3000	5	9.53	3056.76	7.05	0.29	0.08	0.85	0.01
M9	3000	9	9.27	2570.59	7.11	0.10	0.06	0.59	0.05
M9	3000	13	9.85	2095.81	7.19	0.10	0.04	0.50	0.08
M9	3000	17	9.72	1645.23	7.28	4.51	0.14	0.42	0.10
M9	3000	21	9.71	1541.59	7.31	3.50	0.06	0.26	0.09
M9	3000	25	9.72	1197.49	7.40	1.90	0.05	0.28	0.05
M9	3000	29	NA	932.22	7.35	1.00	0.05	0.16	0.01

Table S3: Phytoplankton species identified in the mesocosms and their size class.

Genus	Species	Taxa	Size class
Flagellates	*sp. 1*	Chlorophyta	Micro
Flagellates	*sp. 2*	Chlorophyta	Micro
Pterosperma	*sp.*	Chlorophyta	Micro
Plagioselmis	*prolonga*	Cryptophyta	Micro
Leucocryptos	*marina*	Cryptophyta	Micro
Leucocryptos	*remigera*	Cryptophyta	Micro
Azadinium	*sp.*	Dinophyta	Micro
Gyrodinium	*estuariale*	Dinophyta	Micro
Gymnodinium	*ostenfeldii*	Dinophyta	Micro
Gymnodinium	*ostenfeldii*	Dinophyta	Micro
Protoperidinium	*bipes*	Dinophyta	Micro
Ceratium	*tripos*	Dinophyta	Micro
Ceratium	*longipes*	Dinophyta	Micro
Ceratium	*fusus*	Dinophyta	Micro
Protoperidinium	*depressum*	Dinophyta	Micro
Dinophysis	*norvegica*	Dinophyta	Micro
Dinophysis	*acuminata*	Dinophyta	Micro
Dinoflagellate	*unknown*	Dinophyta	Micro
Eutreptiella	*braarudii*	Euglenophyta	Micro
Flagellate	*euglena*	Euglenophyta	Micro
Calciopappus	*caudatus*	Haptophyta	Micro
Navicula	*sp.*	Haptophyta	Micro
Skeletonema	*sp.*	Heterokontophyta	Micro
Nitzchia	*longissima*	Heterokontophyta	Micro
Pseudonitzchia	*sp.*	Heterokontophyta	Micro
Chaetoceros	*borealis*	Heterokontophyta	Micro
Chaetoceros	*danicus*	Heterokontophyta	Micro
Coscinodiscus	*sp.*	Heterokontophyta	Micro
Chaetoceros	*decipens*	Heterokontophyta	Micro
Thalassiosira	*sp.*	Heterokontophyta	Micro
Pseudopedinella	*pyriformis*	Heterokontophyta	Micro
Chlamidomonas	*sp.*	Chlorophyta	Nano
Flagellates	*sp. 3*	Chlorophyta	Nano
Flagellates	*sp. 4*	Chlorophyta	Nano
Crypto	*sp. 6*	Cryptophyta	Nano
Emiliania	*huxleyi*	Haptophyta	Nano
Arcocellulus	*sp.*	Heterokontophyta	Nano
Synechococcus	*sp.*	Cyanobacteria	Pico
Picoeucarionts	*sp.*	Picoeucariont	Pico
